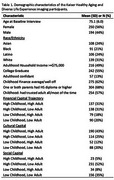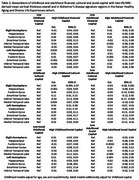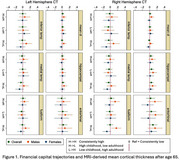# Lifecourse socioeconomic status and MRI‐derived cortical thickness after age 65 in KHANDLE

**DOI:** 10.1002/alz70860_105942

**Published:** 2025-12-23

**Authors:** Rachel Peterson, Pauline Maillard, Nancy X Chen, Batool M. Rizvi, Hilary L. Colbeth, Kazi Sabrina Haq, Kristen M. George, Sarah Tomaszewski Farias, Paola Gilsanz, Elizabeth Rose Mayeda, M. Maria Glymour, Charles Decarli, Rachel A. Whitmer

**Affiliations:** ^1^ University of Montana, Missoula, MT, USA; ^2^ Alzheimer's Disease Research Center, University of California Davis, Sacramento, CA, USA; ^3^ University of California, Davis, Davis, CA, USA; ^4^ University of California, Davis School of Medicine, Sacramento, CA, USA; ^5^ Kaiser Permanente Northern California Division of Research, Pleasanton, CA, USA; ^6^ University of California, Los Angeles Fielding School of Public Health, Los Angeles, CA, USA; ^7^ Boston University School of Public Health, Boston, MA, USA; ^8^ Department of Neurology & Imaging of Dementia and Aging Laboratory, University of California Davis, Sacramento, CA, USA

## Abstract

**Background:**

Adulthood socioeconomic status (SES) is associated with better late‐life brain health but links with lifecourse SES are unclear.

**Method:**

Among Kaiser Healthy Aging Diverse Life Experiences (KHANDLE) participants who completed a brain MRI (*n* = 444), we defined high financial capital in childhood if family finances were “pretty well off” or “about average” and in adulthood if household income was ^3^$75,000; high cultural capital in childhood if at least one parent had a high school diploma, and in adulthood if the participant had a bachelor's degree; high social capital in childhood if the participant had an adult they could trust most/all of the time, and in adulthood if the participant had someone in whom they could confide. SES trajectories were defined as consistently high, high childhood/low adult, low childhood/high adult, and consistently low. Linear regression models estimated associations of childhood and adulthood capital with mean cortical thickness (CT) overall and in AD signature regions. Childhood models adjusted for age at MRI, sex, race/ethnicity and MRI scanner model. Adulthood models additionally adjusted for childhood capital. SES trajectories were examined overall and stratified by sex.

**Result:**

Participants’ mean age was 75.1 (SD=6.0); 56% were female (Table 1). There were no significant differences in CT by financial capital in adulthood or childhood (Table 2). Participants with high (vs. low=reference) childhood cultural capital had greater left hemisphere CT (mean b=0.04 [95%CI=0.01, 0.08]), while those with high (vs. low=reference) adulthood cultural capital had smaller left hemisphere CT (mean b=‐0.04 [95%CI=‐0.07, ‐0.01]). Those with high (vs. low=reference) childhood social capital had smaller left entorhinal CT (b=‐0.08 [95%CI=‐0.01, 0.16), while those with high (vs. low=reference) adulthood social capital had greater CT, especially in the entorhinal cortex (Left b=0.11 [95%CI=0.02, 0.19]; Right b=0.09 [95%CI=0.01, 0.18]). Examination of SES trajectories from childhood to late life revealed a pattern whereby men, but not women, with high financial capital at any point in the lifecourse (ref.=consistently low) trended toward greater left CT (Figure 1).

**Conclusion:**

High childhood cultural capital is an important protective factor for late‐life brain health. Sex‐specific patterns of SES trajectories may provide insights for targeted brain health promotion.